# Pharmacological Neuroenhancement in the Field of Economics—Poll Results from an Online Survey

**DOI:** 10.3389/fpsyg.2016.00520

**Published:** 2016-04-19

**Authors:** Pavel Dietz, Michael Soyka, Andreas G. Franke

**Affiliations:** ^1^Department of Physical Activity and Public Health, Institute of Sport Science, University of GrazGraz, Austria; ^2^Private Clinic Meiringen, Clinic for Psychiatry and PsychotherapyMeiringen, Switzerland; ^3^Department of Psychiatry and Psychotherapy, Ludwig Maximilian UniversityMunich, Germany; ^4^Department of Social Work and Education, University of NeubrandenburgNeubrandenburg, Germany

**Keywords:** neuroenhancement, drugs, misuse, economy, addiction, survey

## Abstract

**Introduction:** The use of over-the-counter, prescription, and illicit drugs to increase attention, concentration, or memory—often called (pharmacological) neuroenhancement—shows a broad range of prevalence rates among students. However, very little data is available on neuroenhancement among employed persons. The aim of this study was to provide first data on substance use for neuroenhancement among readers of the German “Handelsblatt” coming from the field of economics.

**Methods:** Readers of the online edition of the Handelsblatt, a leading print and online medium for the field of economics, were invited to participate in a survey via a link on the journal homepage to complete a web-based questionnaire. Within the questionnaire, participants were asked for their gender, current age, current professional status, hours of work per week, prevalence rates of substance use for the purpose of neuroenhancement as well as for reasons of its use. Binary regression analyses with stepwise forward selection were used to predict the dependent variables “use of illicit and prescription drugs for neuroenhancement” (yes/no), “use of over-the-counter drugs for neuroenhancement” (yes/no), and “use of any drug for neuroenhancement” (yes/no).

**Results:** A total of 1021 participants completed the anonymous survey. Lifetime prevalence for the use of any drug for neuroenhancement was 88.0% and for the use of illicit and prescription drugs for neuroenhancement 19.0%. Reasons and situations that predicted neuroenhancement with illicit and prescription drugs were “curiosity,” “to enhance mood,” ”for a confident appearance,” “stress/pressure to perform,” and “deadline pressure.”

**Discussion:** The study shows that neuroenhancement with drugs is a widespread and frequent phenomenon among people belonging to the professional field of economics. Given in the literature that the use of drugs, especially prescription, and illicit drugs, may be associated with side effects, the high epidemic of drug use for neuroenhancement also shown in the present paper underlines the new public health concern of neuroenhancement.

## Introduction

A 2008 publication of survey results about the use of cognition-enhancing drugs among the readers of *Nature* introduced a phenomenon called neuroenhancement to the scientific world (Maher, 2008). Pharmacological neuroenhancement is frequently defined as the use of any drug to enhance vigilance, attention, concentration, memory, mood, self-confidence, or self-expression without medical need (Greely et al., 2008; Franke et al., 2014). Different terms have been used such as “pharmaceutical/ pharmacological cognitive enhancement,” “mood enhancement,” “academic performance enhancement,” “academic doping,” “cosmetic neurology” etc. to describe the above mentioned aim emphasizing the different cognitive and/or non-cognitive domains and contexts of the use (Chatterjee, 2004; Lucke et al., 2011; Partridge et al., 2011; Fond et al., 2015; Franke et al., 2015b; Sahakian and Morein-Zamir, 2015).

The *Nature* poll assessed the use of Ritalin® (methylphenidate, MPH), Provigil^®;^ (modafinil), and beta blockers for neuroenhancement and found that one fifth of the 1400 participants had used at least one of these drugs to improve their focus, concentration or memory without medical need. The most popular of the assessed drugs was Ritalin® and the most frequent reason for taking the drugs was to improve concentration (Maher, 2008). However, the online poll was then criticized by some authors in the *Nature* blog e.g., regarding the methodology (e.g., participation bias).

An elaborative systematic review by Wilens and colleagues had already shown a past-year prevalence of 5-35% for the general misuse of stimulants among college students (Wilens et al., 2008). Meanwhile, numerous studies from different countries have examined neuroenhancement among students and shown a broad range of prevalence rates for neuroenhancement (between 1 and 20%), depending on the drugs assessed and the survey methods used (McCabe et al., 2005; Boyd et al., 2006; Teter et al., 2006; Franke et al., 2011a; Dietz et al., 2013a; Maier et al., 2013; Webb et al., 2013). The most recent survey among students “shows that prescription drugs, illicit drugs, and lifestyle drugs are, respectively, used by 1.7, 1.3, and 45.6% of the sample” (Schelle et al., 2015).

Surveys about the use of drugs for neuroenhancement in the workplace are very rare. A recent survey among surgeons by Franke and colleagues found a lifetime prevalence rate of 9% for the use of prescription and illicit drugs, 13% for caffeine tablets, and 24% for caffeinated drinks (which describes the mainly German term “Energy drinks” included in the upper most cases caffeine and taurine, excluding cola drinks; Franke et al., 2013, 2015a). Using a technique for increased confidentiality during the assessment (randomized response technique, RRT Campbell, 1987; Moshagen et al., 2010), the group found a lifetime prevalence rate for prescription and illicit drugs of 20% (Franke et al., 2013). A survey by the German DAK found a lifetime prevalence rate of 3.3% for “neuroenhancement” and 4.7% for “neuroenhancement to increase mood or to reduce anxiety and nervousness” [Deutsche Angestelltenkrankenkasse (DAK), 2015]. The KOLIBRI study found a last-year prevalence of 1.5% for all prescription drugs (beta-blockers, stimulants, MPH, antidementia drugs, antidepressants, and modafinil) and 1.0% of which was attributed to antidepressants [Robert-Koch-Institut (RKI), 2011]. The most recent survey concerning neuroenhancement in the workplace using an anonymizing technique revealed a 12-month prevalence rate of 15.4% for the use of prescription drugs among 1186 employed persons (mostly teachers) in Jordan (Wolff et al., 2015). Furthermore, a survey study by Franke and colleagues in 3300 surgeons showed that reducing fatigue, working the night shift and excessive work hours were frequent reasons for using PN substances. This seems to show that coping with unfavorable working conditions such as “stress” are important reasons for using PN drugs (Franke et al., 2013, 2015a). Among students, stress periods in the scope of preparation for exams are associated with the use of PN drugs Burgard et al., 2013). However, among teachers, the willingness of using PN drugs was shown to be low (Sattler et al., 2013; Wiegel et al., 2015). The recent DAK study among 5000 employees showed lifetime prevalence rates of 3.3% for “neuroenhancement” and 4.7% for “neuroenhancement to increase mood or to reduce anxiety and nervousness” [Deutsche Angestelltenkrankenkasse (DAK), 2015].

Numerous studies with divergent study designs have been performed with the group of drugs considered to be neuroenhancers (Mehlman, 2004; De Jongh et al., 2008) to assess the efficacy of the candidate drugs (Solomon et al., 2002; Wesensten et al., 2002; Yesavage et al., 2002; Breitenstein et al., 2004; Killgore et al., 2008, 2009). Overall, the group of stimulants—methylxanthines, e.g., caffeine, and amphetamines, e.g., MPH—seems to have broad pro-cognitive effects on simple cognitive domains that can be affected in fatigued persons. Furthermore, some studies indicate pro-cognitive effects on certain higher cognitive domains, such as particular memory domains. In addition, euphoric effects (in the scope of mood neuroenhancement) of amphetamines have to be considered. (Repantis et al., 2010a,b; Kelley et al., 2012; Franke et al., 2014).

The present web-based study design was inspired by the above mentioned *Nature* poll with the aim to raise data from persons who work in the field of economics or economic-related studies. Therefore, the study assessed for the first time the use of potential neuroenhancers based on an online poll posted on the homepage of the “Handelsblatt” in order to capture data of their readers.

We hypothesized that the prevalence rates of drug use for neuroenhancement within the field of economics as well as associated factors would be similar to previous studies in students, physicians and scientists.

## Materials and methods

The present survey was designed on the basis of the *Nature* poll by Brendan Maher (Maher, 2008). We chose to advertise the survey in the German “*Handelsblatt*,” a print and online medium for people working in the field of economics. The *Handelsblatt*, which until 2005 was part of the company that publishes the *Wall Street Journal* in the US, is the leading economics journal in German-speaking countries; its print run in 2014 was 120,000 and in Germany it is the most cited print medium for economics (Handelsblatt). Readers of the online edition of the *Handelsblatt* were invited to participate in the present survey via a link on the homepage of the journal (www.Handelsblatt.com)^1^.

### Data acquisition

To ensure a high degree of privacy and anonymity, the survey was designed as an online poll with eight closed questions about participants' characteristics and their patterns of drug use for neuroenhancement. The survey was open for participation during the last 2 weeks of December 2014. Before starting the survey, readers read a short introductory paragraph that introduced them to the topic of neuroenhancement and explained the survey. Then, after answering questions about their gender, current age, current professional status, and hours of work per week, participants were asked if they would at all consider using (a) legal over-the-counter drugs (OTC drugs) (b) prescription drugs, or (c) illicit drugs for neuroenhancement or (d) no drugs for enhancement at all. Directly afterwards, participants were asked to indicate any substances they had already used for neuroenhancement by completing a table that listed OTC drugs and prescription and illicit drugs as well as drinks known to be frequently used for neuroenhancement by students (Mehlman, 2004; De Jongh et al., 2008; Franke et al., 2011b, 2014; Dietz et al., 2013b; Schelle et al., 2015) and surgeons (Franke et al., 2013, 2015a): coffee, energy/caffeinated drinks, caffeine tablets, cola drinks, Ginkgo biloba, Ritalin®, Adderall®, modafinil, ecstasy, ephedrine, cocaine, crystal meth, illicit amphetamines, and antidepressants. Subsequently, participants were asked which of the following situations and reasons were associated with their use of the aforementioned drug or drugs for neuroenhancement (multiple responses were possible): (a) curiosity, (b) stress/pressure to perform, (c) tiredness, (d) to enhance mood, (e) for a confident appearance, (f) deadline pressure, (g) other; these categories were identified in previous studies in students as reasons and situations commonly associated with the use of drugs for neuroenhancement.

In order to exclude from further analysis participants who used certain prescription drugs to treat a disease, participants were asked if they suffered from attention-deficit-hyperactivity disorder (ADHD) or depression. Participants who stated to “have been diagnosed” with ADHD or depression were excluded.

### Data analysis

Data were collected in a database connected directly to the survey questionnaire. Statistical analyses were performed with SPSS for Windows, version 22.0. Binary regression analysis with stepwise forward selection was used to predict the dependent variables “use of illicit and prescription drugs for neuroenhancement” (yes/no), “use of OTC drugs for neuroenhancement” (yes/no), and “use of any drug for neuroenhancement” (yes/no). Continuous variables (age, hours of work per week) were dichotomized by mean. Results are given as means and standard deviations (SD), prevalence rates (%), or odds ratios (OR) with Clopper-Pearson confidence intervals (95% CI) and *p*-values.

### Ethics statement

The study was performed according to the Declaration of Helsinki. Participants gave informed consent by clicking on a button after reading the short introductory paragraph and by pressing the button “done” at the end of the survey. The study was approved by the responsible ethics committee (Greifswald; approval no. BB 095/14).

## Results

In total, 1021 readers of the journal *Handelsblatt* participated in the survey. Among the participants, 6.6% (*n* = 67) reported to have been diagnosed with ADHD or depression and had to be excluded from further analysis. Therefore, the data of 954 participants were analyzed. Most of the participants were male (82.7%), with a mean age of 36.3 years. Approximately one-third of the participants rated themselves as belonging to “middle management” (32.4%). Participants worked aF mean of 48.7 h per week. Table 1 gives a detailed description of participant characteristics.

**Table 1:** **Basic characteristics of the survey participants**.

**Characteristic**	**Frequency or range, mean and standard deviation of characteristic among participants (*N* = 954)**
**Gender**	82.7% male (*n* = 767)
	17.3% female (*n* = 160)
**Age**, y (mean, SD)	17–71 (36.3, 11.2)
**CURRENT PROFESSION**	
“Simple” employee	28.8% (*n* = 270)
Middle management	32.4% (*n* = 304)
Top management	9.6% (*n* = 90)
Freelancer	12.5% (*n* = 117)
Public official	1.5% (*n* = 14)
Student studying economics	15.0% (*n* = 141)
Not working	0.3% (*n* = 3)
**Hours of work per week**, h (mean, SD)	4–100 (48.7, 11.1)

Regarding the question of whether the participants would consider using any substance for neuroenhancement, 40.0% (*n* = 372) answered “yes, an OTC substance,” 12.6% (*n* = 117) answered “yes, a prescription drug” and 8.3% (*n* = 77) answered “yes, an illicit drug.”

Among all participants, 88.0% (*n* = 831) had used one of the listed substances (see Materials and Methods Section) for neuroenhancement purposes at least once in their life (lifetime prevalence): lifetime prevalence was 87.5% (*n* = 824) for the use of any OTC drug for neuroenhancement and 19.0% (*n* = 171) for the use of any prescription or illicit drug for neuroenhancement. Among the group of OTC drugs and drinks the most commonly used substance was coffee (lifetime prevalence: 77.1%, *n* = 723), followed by cola drinks (Coca-Cola®, etc.; lifetime prevalence: 56.6%, *n* = 521; see Table 2). The most frequently used drugs among the group of prescription and illicit drugs were antidepressants, with a lifetime prevalence of 7.2% (*n* = 65) and a period prevalence rate in the last month of 2.0% (*n* = 18); during the last month only cocaine was used more frequently (2.3%, *n* = 20; Table 2). For more detailed information about prevalence rates, see Table 2.

**Table 2:** **Prevalence rates for use of substances for neuroenhancement among survey participants (*N* = 954)**.

**Use of any surveyed substance**	**Never used**	**Used**			
		**Total responses**	**Within the last month**	**Within the last 12 months**	**More than 12 months ago**
**OTC DRUGS/DRINKS**					
Coffee	22.9%	77.1%	43.5%	7.6%	26.0%
	(*n* = 215)	(*n* = 723)	(*n* = 408)	(*n* = 71)	(*n* = 244)
Energy/caffeinated drinks	53.3%	46.7%	21.0%	13.6%	12.0%
	(*n* = 489)	(*n* = 428)	(*n* = 193)	(*n* = 125)	(*n* = 110)
Caffeine tablets	74.9%	25.1%	5.8%	6.3%	13.0%
	(*n* = 669)	(*n* = 224)	(*n* = 52)	(*n* = 56)	(*n* = 116)
Cola drinks (e.g., Coca-Cola^®;^, etc.)	43.4%	56.6%	31.2%	11.5%	13.9%
	(*n* = 399)	(*n* = 521)	(*n* = 287)	(*n* = 106)	(*n* = 128)
Ginkgo biloba	89.4%	10.6%	2.7%	3.7%	4.1%
	(*n* = 788)	(*n* = 93)	(*n* = 24)	(*n* = 33)	(*n* = 36)
**PRESCRIPTION AND ILLICIT DRUGS**					
Ritalin^®;^	94.9%	5.1%	1.5%	1.9%	1.7%
	(*n* = 852)	(*n* = 45)	(*n* = 13)	(*n* = 17)	(*n* = 15)
Adderall^®;^	96.7%	3.3%	0.8%	0.8%	1.7%
	(*n* = 853)	(*n* = 29)	(*n* = 7)	(*n* = 7)	(*n* = 15)
Modafinil	97.7%	2.3%	0.9%	0.5%	0.9%
	(*n* = 857)	(*n* = 20)	(*n* = 8)	(*n* = 4)	(*n* = 8)
Ecstasy	95.6%	4.4%	1.6%	0.9%	1.9%
	(*n* = 843)	(*n* = 39)	(*n* = 14)	(*n* = 8)	(*n* = 17)
Ephedrine	94.7%	5.3%	1.6%	1.4%	2.3%
	(*n* = 829)	(*n* = 46)	(*n* = 14)	(*n* = 12)	(*n* = 20)
Cocaine	93.5%	6.5%	2.3%	1.3%	3.0%
	(*n* = 820)	(*n* = 57)	(*n* = 20)	(*n* = 11)	(*n* = 26)
Crystal meth	98.2%	1.8%	0.6%	0.5%	0.8%
	(*n* = 857)	(*n* = 16)	(*n* = 5)	(*n* = 4)	(*n* = 7)
Illicit AMPH	93.0%	7.0%	1.9%	1.9%	3.2%
	(*n* = 826)	(*n* = 62)	(*n* = 17)	(*n* = 17)	(*n* = 28)
Antidepressants	92.8%	7.2%	2.0%	2.5%	2.7%
	(*n* = 839)	(*n* = 65)	(*n* = 18)	(*n* = 23)	(*n* = 24)

*AMPH, amphetamines; “ever,” within the last month + within the last 12 months + more than 12 months ago; OTC drugs, over-the-counter drugs*.

The most frequently mentioned situations or reasons associated with using any drug for neuroenhancement including OTC and prescription and illicit drugs were “tiredness” (79.4%, *n* = 533), “stress/pressure to perform” (41.7%, *n* = 280), and “to enhance mood” (22.2%, *n* = 149). The rates for “deadline pressure,” “curiosity,” “for a confident appearance,” and “others” were 17.3% (*n* = 116), 12.2% (*n* = 82), 11.8% (*n* = 79), and 11.5% (*n* = 77), respectively; Table 3 gives further details about situations or reasons associated with the use of OTC drugs and prescription and illicit drugs.

**Table 3:** **Reasons and situations associated with the use of drugs for neuroenhancement**.

	**OTC drugs (*N* = 669)**	**Prescription and illicit drugs (*N* = 155)**	**OTC drugs + prescription and illicit drugs (*N* = 671)**
Curiosity	12.1%	31.0%	12.2%
	(*n* = 81)	(*n* = 48)	(*n* = 82)
Stress, pressure to perform	41.7%	60.6%	41.7%
	(*n* = 279)	(*n* = 94)	(*n* = 280)
Tiredness	79.4%	72.3%	79.4%
	(*n* = 531)	(*n* = 112)	(*n* = 533)
To enhance mood	22.1%	45.8%	22.2%
	(*n* = 148)	(*n* = 71)	(*n* = 149)
For a confident appearance	11.7%	29.0%	11.8%
	(*n* = 78)	(*n* = 45)	(*n* = 79)
Deadline pressure	17.2%	28.4%	17.3%
	(*n* = 115)	(*n* = 44)	(*n* = 116)
Others	11.5%	16.8%	11.5%
	(*n* = 77)	(*n* = 26)	(*n* = 77)

*OTC drugs, over-the-counter drugs*.

Binary logistic regression revealed five independent variables that predict the use of illicit and prescription drugs for neuroenhancement: “curiosity,” “stress/pressure to perform,” “to enhance mood,” “for a confident appearance,” and “deadline pressure” (see Table 4). The dichotomized independent variables “age” and “hours of work per week” as well as each of the items “current profession,” “using OTC drugs for neuroenhancement,” “gender,” and “tiredness” did not significantly predict the use of illicit and prescription drugs for neuroenhancement. Only two independent predictor variables, “tiredness” and “stress/ pressure to perform,” were found for OTC drug use for neuroenhancement and for the use of any substance for neuroenhancement (including OTC, illicit and prescription drugs), respectively (see Table 4).

**Table 4:** **Predicting factors for the use of substances for neuroenhancement**.

**Predictor**	**OR (95% CI)**
**USE OF ILLICIT AND PRESCRIPTION DRUGS FOR EUROENHANCEMENT**	
Curiosity	4.79^***^ (2.75–8.33)
To enhance mood	2.72^***^ (1.68–4.42)
For a confident appearance	2.69^**^ (1.47–4.91)
Stress/ pressure to perform	1.83^*^ (1.15–2.91)
Deadline pressure	1.79^*^ (1.03–3.11)
**USE OF OTC DRUGS FOR NEUROENHANCEMENT**	
Tiredness	22.44^***^ (4.68–107.64)
Stress/pressure to perform	10.15^***^ (1.24–83.01)
**USE OF ANY SUBSTANCE FOR NEUROENHANCEMENT**	
Tiredness	45.24^***^ (5.46–374.77)
Stress/pressure to perform	9.59^***^ (1.13–81.12)

Odds ratios for the dependent variables “use of illicit and prescription drugs for neuroenhancement,” “use of OTC-drugs for neuroenhancement,” and “use of any substance for neuroenhancement” and each predictor variable (stepwise, forward regression). Levels of significance:

p < 0.05^*^;

p < 0.01^**^;

p < 0.001^***^;

*(CI, confidence interval; OR, odds ratio); OTC drugs, over-the-counter drugs*.

The areas under the receiving operation curve (ROC) of the overall regression models were 77.2% for “use of illicit and prescription drugs for neuroenhancement,” 85.5% for “use of OTC drugs for neuroenhancement,” and 88.1% for “use of any substance for neuroenhancement,” respectively.

## Discussion

This study used an anonymous web-based questionnaire to investigate for the first time the use of drugs for neuroenhancement among the “Handelsblatt” readership working or at least studying in the field of economics. The study shows that the prevalence rates of neuroenhancement in the surveyed participants within the field of economics are similar to those in other highly demanding fields. Although, the associated reasons and situations are somewhat similar, non-cognitive reasons for neuroenhancement are much more important in our participants than among the previously studied groups (students, surgeons).

Among the large number of papers on neuroenhancement, one of the most extensive studies (in 8000 students) found lifetime prevalence rates of 5% for prescription drugs and 5% for so-called “soft enhancement” (use of vitamins, homeopathic drugs, “herbal” substances, caffeine, etc.), i.e., significantly lower rates than that in our study (40.0%; Middendorff et al., 2012).

There is a paucity of data about neuroenhancement among the general population and employed persons in particular. For their 2015 health report, a German health insurance company (DAK) re-assessed a representative panel of 5000 participants aged between 20 and 50 years [Deutsche Angestelltenkrankenkasse (DAK), 2015]. The online study had a response rate of 49.1% and found lifetime prevalence rates of 3.3% for “neuroenhancement” and 4.7% for “neuroenhancement to increase mood or to reduce anxiety and nervousness” [Deutsche Angestelltenkrankenkasse (DAK), 2015]. Cognitive neuroenhancement was more frequent among men, and mood neuroenhancement, as well as reduction of anxiety and nervousness, more frequent among women [Deutsche Angestelltenkrankenkasse (DAK), 2015]. This finding is in contrast to our study results. We found significantly higher prevalence rates and no differences between men and women regarding special patterns of substance use which may be due to the inequal preponderance in our study in contrast to the DAK study. However, in line with the DAK study we found higher prevalence rates for mood neuroenhancement than for cognitive neuroenhancement, which we consider to be one of the most important findings of our study.

The Kolibri study evaluated 6000 participants from the general public and found a last-year prevalence of 1.5% for all prescription drugs (beta-blockers, stimulants, MPH, antidementia drugs, antidepressants and modafinil), 1.0% of which was attributed to antidepressants [Robert-Koch-Institut (RKI), 2011]. The relatively high prevalence rates for the use of potential mood-enhancing substances concur with our findings.

One German web-based study used vignettes to examine the prevalence rate of neuroenhancement among university teachers and their willingness to use drugs for neuroenhancement and found both to be low (Sattler et al., 2013; Wiegel et al., 2015). However, teachers are not comparable with the participants surveyed in this study.

The *Nature* poll mentioned above found a lifetime prevalence rate of 20% for the use of beta-blockers, methylphenidate (MPH; Ritalin®), and modafinil (Maher, 2008) which is nearly the same as that found among the participants of the present survey (19.0% lifetime prevalence rate for prescription and illicit drug use). Ritalin® was the most commonly used drug in the *Nature* poll, and in our study lifetime prevalence rates were highest for illicit amphetamines. Considering that MPH belongs to the group of amphetamines and Ritalin® was the third most frequently used prescription/illicit drug in our survey, the results are again comparable. Beyond the question of the frequency of use for neuroenhancement, Maher found that the most popular reasons for the use of the three assessed drugs were to improve concentration, improve focus for a specific task, and counteract jetlag (Maher, 2008). Interestingly, these reasons do not overlap with those found in our study, which identified curiosity, mood neuroenhancment, and confident appearance as the three most important reasons for neuroenhancement.

Franke et al. studied 3300 surgeons and found a lifetime prevalence rate for the use of prescription and illicit stimulants of 8.9% when participants completed a direct questioning survey (paper-and-pencil questionnaire) and 19.9% when the specialized anonymizing survey technique RRT was applied (Franke et al., 2013, 2015a). Furthermore, lifetime, past-year, past-month, and past-week prevalence rates for coffee were 66.8, 61.9, 56.9, and 50.5%, for caffeinated drinks 24.2, 15.4, 9.9, and 6.1%, and for caffeine tablets 12.6, 5.9, 4.7, and 3.8%, respectively. Although, prevalence rates for coffee were similar to those in the present study, those for caffeinated/energy drinks and caffeine tablets were significantly lower (Franke et al., 2015a). Prevalence rates for (psycho-) stimulants were higher among surgeons than in our study (Franke et al., 2013).

Among surgeons, reducing fatigue (54.3%), working the night shift (32.2%), and overly long and excessive work hours (31.7%) were the most frequent reasons for using caffeine (Franke et al., 2013). This finding could only be partially confirmed in the present study, which identified tiredness, stress/ pressure to perform, and mood neuroenhancement as the three most important reasons for using caffeine.

When Franke and colleagues compared the use of prescription and illicit drugs by surgeons for cognitive neuroenhancement with the use of antidepressants for mood neuroenhancement, which was a separate category in the study, they found lifetime prevalence rates of 8.9% for the former and only 2.4% for the latter (Franke et al., 2013). Our study found much higher prevalence rates for antidepressant use without medical need (7.2%, *n* = 65), i.e., the rate was three-fold higher among the present participants. The reason for this discrepancy is unclear. Therefore, future studies should address in detail the reasons why participants use drugs for the purpose of neuroenhancement and for mood enhancement.

The question of anonymity and privacy when using a survey about potentially stigmatizing issues is complex. Empirical social science has shown a tendency for people to answer with socially desirable answers when asked about sensitive or stigmatizing issues (e.g., own thievery; Schnell et al., 1992). This aspect is important when asking participants about their use of neuroenhancement drugs which, at least in the case of misusing prescription drugs and using illicit drugs, is potentially punishable. Therefore, other studies using the RRT—which is only one among a variety of techniques for the assessment of socially undesirable behavior (Campbell, 1987; Moshagen et al., 2010; Wolff et al., 2015)—to assess the use of antidepressants among surgeons, found prevalence rates of 19% for cognitive neuroenhancement and 15% for mood neuroenhancement. Prevalence rates for cognitive neuroenhancement are comparable to our results; however, prevalence rates for mood neuroenhancement are considerably higher. Comparable prevalence rates for neuroenhancement may imply that an online poll gives a subjective feeling of anonymity and privacy similar to the RRT, perhaps because (more or less) nobody can trace answers back to the participants.

Taken together, the high discrepancy between the direct questioning and RRT results for antidepressants and the high prevalence rates for the use of antidepressants found in the present study could mean that mood neuroenhancement and not cognitive neuroenhancement is the “core enhancement” phenomenon. Because stimulants also have euphoric effects, they are perhaps being used for both purposes. An as yet underestimated reason could be to feel more self-confident and to enhance mood. Even though this assumption is highly speculative, it is supported by the present study and may therefore warrant further investigation.

In addition to the aspects mentioned above, some factors should be addressed that limit the explanatory power of this study. As with every survey study, one can discuss the suitability of the content and length of the questionnaire and the likelihood of complete participation. Our questionnaire, inspired by the online *Nature* poll (Maher, 2008) was designed to increase the likelihood of participation and the completeness of participants' responses at the expense of losing a large amount of information because of its brevity. Therefore, the authors designed the questionnaire on the basis of the available neuroenhancement literature to be brief but nevertheless informative. A web-based survey has an inherent risk of participation bias because one cannot control for participants' experiences, opinions, personal characteristics, and subjective aims when answering the questions. Nevertheless, we considered such a survey the best way to collect “real life” data and gain insight into the fast-paced field of economics.

The main strength of this study, its high degree of anonymity and privacy, is also the cause of one of its most **limiting factors**, the participation bias. Because the present study was voluntarily conducted as an online poll—to guarantee the highest degree of anonymity and privacy—in the *Handelsblatt*, readers of the online version of this specific journal were able to participate. However, because of the possibility to distribute links via social media networks, it cannot be guaranteed that only readers of the *Handelsblatt* participated. This may explain the fact, that the group of participants is more or less heterogeneous. Means to reduce participation bias and to control for participation (e.g., via participation codes, etc.) leads to reduced anonymity and privacy and were not considered by the authors in order to receive honest answers. This major limitation was of similar concern in the *Nature* poll by Brendan Maher.

Beyond that, the online version is widely read and only ~1000 individuals participated, which may not be representative of only those working in the field of economics. Furthermore, we have no information about possible specific characteristics of the individuals who did not participate. Therefore, we do not know whether those individuals would have increased or decreased the prevalence rate for neuroenhancement or shown additional predictors. Online surveys cannot control for such a disproportion and response bias.

In sum, the study results presented here show that drug use to increase cognition, enhance mood, improve confidence, or cope with stress and pressure seems to be a widespread phenomenon within the field of economics. Taken together, the results of this and previous studies indicate that the findings of studies in high school, college, and university students may also be valid in employed persons. Findings in employed persons such as surgeons, individuals working in the professional field of economics, and natural scientists demonstrate that neuroenhancement has become a widespread phenomenon. Both are associated with health concerns, because any (mis-) use of drugs may have adverse effects on mental and physical health. For example, any stimulant is associated with the risk of cardiovascular events, hypertonia, tachycardia, and even sudden cardiac death (e.g., Kumar, 2008; Ali et al., 2015; Vetter et al., 2015). Such events may be more frequent when drugs are misused without a physicians' prescription or are mixed with other drugs. Additionally, drug misuse can lead to addiction and be a gateway for the use of other—more harmful or illicit—drugs (Kandel, 2002; Dietz et al., 2013b, 2016). Taking these negative aspects together and considering the high prevalence rates of drug use for neuroenhancement, we think that the creation of prevention programs and related educational material is of great public health relevance. A large journal with an online version like the *Handelsblatt*, which is read by a potential at-risk population, could be used to spread information and material about such programs. Additionally, the predictors for drug use for neuroenhancement identified in the present study may help to create well-directed prevention programs and education material tailored more closely to individual needs. As the metric scaled variables in the present study were dichotomized for analyses, one implication for future studies would be to provide more individual profiles of drug users, addressing more specific demographic characteristics, rather than the dichotomized method used in the current study.

## Author contributions

AF made substantial contributions to the conception and design of the study, the data acquisition, analysis, and interpretation of data, and the preparation of the manuscript. MS made substantial contributions to the interpretation of data and the preparation of the manuscript. PD made substantial contributions to the analysis and interpretation of data and the preparation of the manuscript. All authors proofread and accepted the final version of the manuscript.

### Conflict of interest statement

The authors declare that the research was conducted in the absence of any commercial or financial relationships that could be construed as a potential conflict of interest.

## Footnotes

^1^According to a personal communication from the Handelsblatt and www.Handelsblatt.com (accessed in april 2015) and www.ivw.eu (accessed in april 2015).
